# Evolution of high-sensitivity troponin-T and echocardiography parameters in patients undergoing high efficiency on-line hemodiafiltration versus conventional low-flux hemodialysis

**DOI:** 10.1371/journal.pone.0223957

**Published:** 2019-10-22

**Authors:** Isabelle Ethier, Dominique Auger, Martin Beaulieu, Ewa Wesolowska, Renée Lévesque

**Affiliations:** 1 Department of Nephrology, Centre Hospitalier de l’Université de Montréal, Montreal, Quebec, Canada; 2 Department of Cardiology, Centre Hospitalier de l’Université de Montréal, Montreal, Quebec, Canada; 3 Department of Biochemistry, Centre Hospitalier de l’Université de Montréal, Montreal, Quebec, Canada; Universidade Nove de Julho, BRAZIL

## Abstract

**Background and objectives:**

On-line hemodiafiltration (HDF) has been associated with better inflammatory markers profile and survival than low-flux hemodialysis (HD). This study aimed at determining the effect of HDF vs HD on hs-TnT and echocardiography parameters evolution at one year follow-up.

**Method:**

Patients were randomized from 2007 to 2013 to HD or HDF in accordance with the CONvective TRAnsport STudy protocol initially as part of the Montreal cohort and subsequently as part of a local cohort. Pre-dialysis hs-TnT were analyzed at baseline and 1-year follow-up.

**Results:**

A total of 54 HDF patients and 59 HD patients were included. At baseline, median hs-TnT value was 49 ng/L (IQR 31–89) in the HDF group vs. 60 ng/L (36–96) in the HD group (p = 0.370). At one year follow-up, median hs-TnT remained stable in the HDF group (p = 0.707 vs. baseline), but significantly increased to 62 ng/L (40–104) in the HD group (p = 0.021 vs. baseline). The median variation (delta) in hs-TnT values was -3 ng/L (IQR -7-+8) in the HDF group vs. +8 ng/L (-5 -+25) in the HD group (p = 0.042). In the HDF group, LVEF increased from 60.0% (IQR 55.0–65.0) at baseline to 65.0% (60.0–65.5) at 1-year follow-up (p = 0.040) whereas it remained stable in the HD group (LVEF of 60.0% [IQR 55.0–65.0] at baseline and 65.0% [55.0–65.0] at 1-year follow-up [p = 0.312]).

**Conclusions:**

High-efficiency HDF is associated with stability in hs-TnT values, whereas low-flux HD is associated with significant increase in hs-TnT levels.

## Introduction

Patients in end stage kidney disease (ESKD) have an increased risk for morbidity and mortality, and the main cause of death is cardiovascular disease [1]. On-line hemodiafiltration (HDF) has been associated with better atherosclerosis-related inflammatory markers profile than conventional low-flux hemodialysis (HD) [2–3]. Large randomized controlled trials suggest that HDF has a beneficial effect on survival when higher convection volumes are provided [4–7]. The HDF Pooling Project Investigators combined data from four recent RCTs and showed a decrease in both all-cause (14%) and cardiovascular (23%) mortality with HDF [8]. The highest benefit was obtained with convection volumes >23L. However, data on biologic markers and cardiac morphology were not compared.

Moreover, it has been shown that, in hemodialysis patients, troponin T is independently related to left ventricular mass and is a good predictor of all-cause and cardiovascular mortality [9–11]. Increasing troponin T concentration over time has also been linked to all-cause mortality in hemodialysis patients [12]. More recent studies have shown that high-sensitivity troponin T (hs-TnT) is related to all-cause and cardiovascular mortality in end-stage renal disease patients, with or without cardiac disease [13–14] and that stable asymptomatic hemodialysis patients have elevated hs-TnT at baseline [15–16]. Other cardiac biomarkers, such as creatine-kinase MB isoenzyme (CKMB), have been evaluated for stratifying cardiovascular risk in dialysis patients and associated with cardiovascular events [17]. Nevertheless, data are conflicting on the effect of dialysis on hs-TNT and CKMB levels [18].

Furthermore, left ventricular mass (LVM) and left ventricular ejection fraction (LVEF) have been repeatedly demonstrated to be relevant predictors of cardiovascular morbidity and mortality in ESKD patients [19]. Studies on the natural trend of these parameters in ESKD have reported either raise or stabilization of LVM over a period of 18–24 months, while the course of EF is to slowly decrease [20]. In a sub-study of the CONTRAST trial, treatment with online HDF failed to demonstrate a difference in LVM and LVEF over time as compared to HD [7]. However, a small randomized trial, showed a tendency to a smaller increase in myocardial mass and a significant increase in LVEF in HDF compared to HD [21].

On-line HDF in our center is conducted with high convection volumes (>24L/session). We therefore aimed at determining the effect of high efficiency on-line HDF vs HD on hs-TnT and CKMB levels, LVM and LVEF at 1-year follow-up. Predictors of hs-TnT levels at 12 months were also evaluated.

## Materials and methods

### Patients and study design

The present study was conducted within the scope of the CONTRAST study [4] and a subsequent local study. The CONTRAST protocol was approved by the local medical ethics committee on June 20^th^ 2006 (www.clinicaltrials.gov; identifier NCT00205556). As part of the CONTRAST study, patients were locally randomized from November 19^th^ 2007 and followed up until December 31^st^ 2010. During the CONTRAST study, our center conducted OL-HDF with the highest convection volume (>24 L/session) of all participating centers. The decision to continue the enrollment after the end of the CONTRAST study was approved on February 28^th^ 2011 by the local medical ethics review board for a subsequent cost-effectiveness local study comparing HDF to low-flux HD (www.clinicaltrials.gov; identifier NCT02374372). The authors confirm that all related trials for this intervention are registered. Written informed consent to continue the study was obtained from all trial participants who were still enrolled by the end of the CONTRAST study (i.e. from December 31, 2010 until November 30, 2015). For newly enrolled patients, written informed consent was also obtained (prior to randomization). Randomization procedure, monitoring, and data collection were done the same way than during the CONTRAST period. During the CONTRAST period, patients were randomized centrally through a computer-based service into a 1:1 ratio for treatment with HDF or low-flux HD using permuted blocks, stratified by center. After the CONTRAST period, the same permuted blocks randomization scheme was used through a local process managed by administrative personnel completely blind to the whole study. Patients were followed up as part of this local cost-effectiveness study until November 30^th^ 2015. Detailed information on study design and conduct, as well as inclusion and exclusion criteria can be found in the original CONTRAST protocol [22] and in the original local cost-effectiveness protocol [23]. During both study periods, patients consented in having blood samples drawn for further analysis of inflammation and cardiovascular biomarkers.

The present study represents a sub-study of the aforementioned cost-effectiveness study. In this study, patients for whom blood samples at baseline could not be analyzed for hs-TnT or who did not get an echocardiography at baseline were excluded. Patients for whom hs-TnT and echocardiography were both missing at 1-year for reasons other than loss to follow-up were also excluded from analysis. Finally, patients with myocardial infarction within 2 months before randomization were excluded (none). As some patients were excluded from the original cohort for this sub-study after randomization, we cannot affirm the complete absence of confounding.

Demographic data were obtained at baseline. Dialysis data at baseline and at 1-year were also recorded. Kt/V for all patients and beta-2-microglobulin reduction rate for patients in HDF were calculated using Bergstrom’s formula [24].

### HD and HDF prescription

Online HDF was performed in the post-dilution mode using 4008 ONLINE system (Fresenius Medical Care, Bad Hamburg, Germany), with Optiflux 200NR (Fresenius Medical Care) dialyser membrane. The target convective volume was arbitrarily set at 6L/h (or 100 ml/min) or the best convective volume achieved by the vascular access. HD patients were treated with synthetic low-flux dialyzers (Optiflux 18NR, Fresenius Medical Care) with Integra (Gambro AB, Lund, Sweden) dialysis system. As per the CONTRAST protocol [22], all patients were treated two to three times per week and had to be stable with a minimum dialysis single-pool Kt/V for urea (spKt/V_urea_) of 1.2 before randomization. Treatment times were fixed at baseline and could be increased if the dialysis urea spKt/V_urea_ was below 1.2. Fluid management was performed according to national and international quality of care guidelines as part of routine patient care, which locally referred to regular clinical assessment by the attending nephrologist on the dialysis unit, without any instrumental support such as bioimpedance. Ultrapure quality of water and dialysis fluids was regularly monitored and maintained during the study.

### Primary and secondary end points

Primary end point was evolution of hs-TnT levels from baseline at 1-year follow-up depending on the type of hemodialysis (HD vs. HDF). Secondary end points were left ventricular mass index (LVMI) and left ventricular ejection fraction (LVEF), as obtained by echocardiography. Evolution of CKMB levels from baseline to 1-year follow-up was also evaluated to further explore the kinetics of cardiac biomarkers with different types of dialysis. Indeed, CKMB’s molecular weight of 82 kD should prevent it from being cleared by both HD and HDF and allow interesting comparison with troponins’ level evolution.

Blood samples were obtained before dialysis session at baseline and at one year, and were analyzed for hs-TnT with Roche Diagnostics STAT assay and for creatine kinase MB-mass (CKMB-mass) with Roche Diagnostics assay on Cobas e411 instrument at the beginning of the present study. Throughout the study, patients also had monthly blood tests as per usual follow-up in the dialysis unit. However, monthly blood tests did not include hs-TnT.

Echocardiography was performed locally by cardiologists, blinded for treatment assignment. Echocardiography was obtained at baseline, before randomization, and at 1-year follow-up. All echocardiographic studies needed to be done less than 24 hours after a dialysis treatment session. All images were acquired on a Vivid 7 or E9 machine (General Electric Healthcare, Chicago, Illinois, USA) in the left lateral recumbent position. LVEF was obtained by the bi-plane Simpson method of disks [25]. LV mass was calculated with the 2D method according to the American Society of Echocardiography guidelines [25] and was indexed to body surface area according to the Mosteller formula [26].

### Statistical analysis

Continuous data are reported as mean ± standard deviation when distributed normally and as median (interquartile range) when not distributed normally. Categorical data are expressed as frequency (percentage). Between groups comparison of patients’ characteristics, hs-TnT values, echocardiography parameters and dialysis data at baseline were performed with T-Test when distributed normally and Mann-Whitney U test when not distributed normally. As distribution was not normal, evaluation of the evolution of hs-TnT and CKMB values at baseline and at 1-year follow-up were performed with appropriate Wilcoxon signed-rank tests. To determine the predictors of hs-TnT levels at 12 months’ follow-up, a general linear regression was used. All significant variables at the univariate analysis were used in a multivariable model, with the enter method, a procedure for variable selection in which all variables in a block are entered in a single step. Two-sided p-values less than 0.05 were considered statistically significant. All analyses were done using SPSS software version 22.0.0, IBM, Chicago, Illinois, USA.

## Results

**Fig 1:**
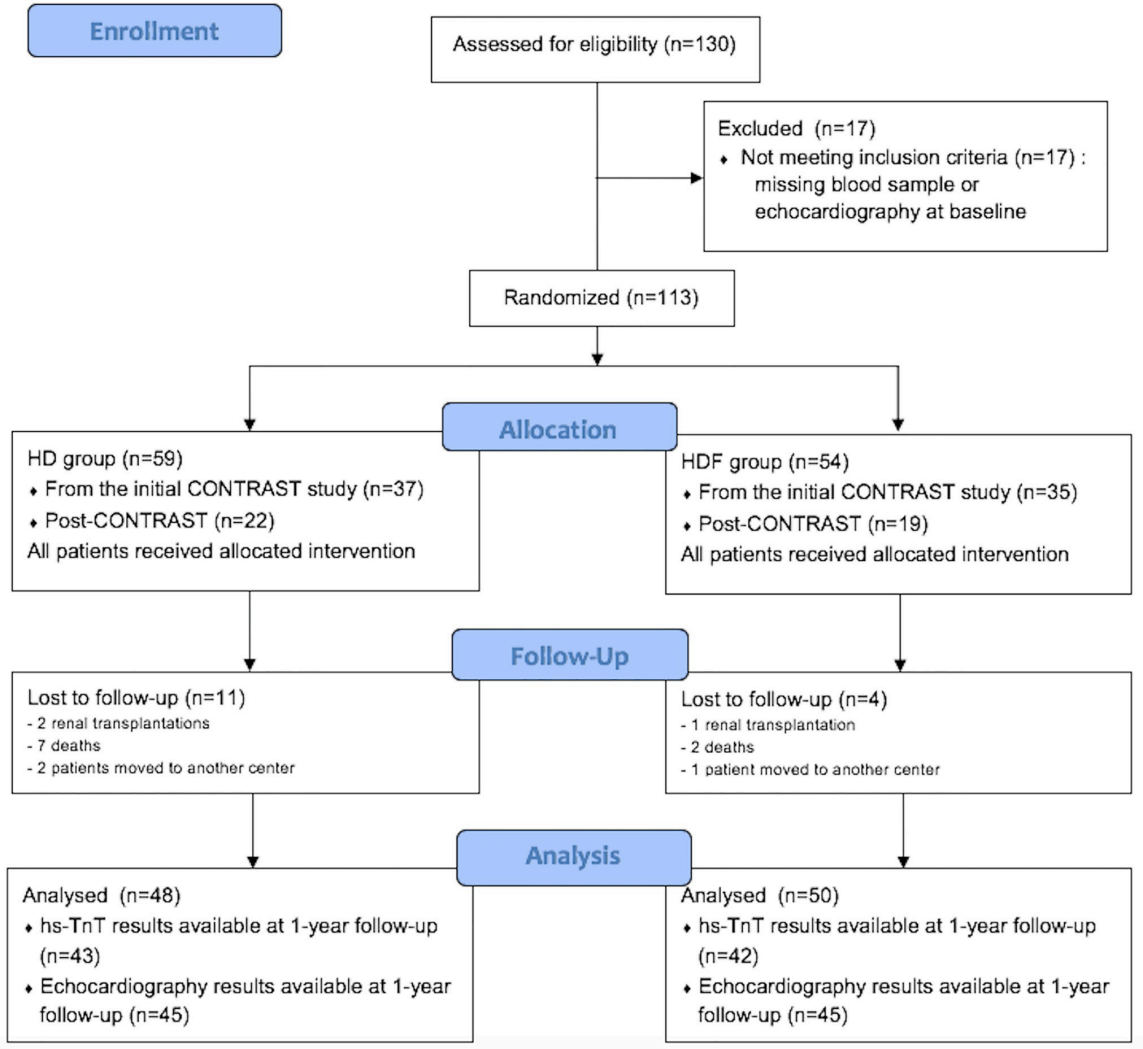
Study profile.

A total of 54 HDF patients (35 as part of the initial CONTRAST study) and 59 HD patients (37 as part of the initial CONTRAST study) were included in the present study. At one year, there were 4 (7%) patients in the HDF group and 11 patients (19%) in the HD group loss to follow-up (Fig 1). Additionally, hs-TnT values at 1-year follow-up were unavailable for 5 patients in the HD group and 8 patients in the HDF group, either because blood samples were not obtained at the time, quantities of blood samples kept were insufficient to analyze or the 1-year follow-up time had not been reached yet at the time of data analysis. Echocardiography data was unavailable for 4 patients in the HDF group and 3 in the HD group, because patients missed their planned appointment (Fig 1). Loss to follow-up and additional missing values left 48 and 50 patients in the HD and HDF groups, respectively, for analysis at 1-year follow-up. At baseline, patients in both groups were comparable in age, gender, comorbidities and use of cardiovascular medication (Table 1).

**Table 1:** Baseline characteristics of the patients.

Variables	HDF (n = 54)	HD (n = 59)	P-value
**Age (years)**	65 ± 15	66 ± 11	0.587
**Male gender**	32 (59%)	40 (68%)	0.434
**Active smoking**	4 (7%)	8 (14%)	0.407
**BMI (kg/m**^**2**^**)**	27 ± 6	28 ± 7	0.940
**CAD**	26 (48%)	31 (53%)	0.390
**Diabetes mellitus**	27 (50%)	34 (58%)	0.266
**Hypertension**	47 (87%)	47 (80%)	0.076
**Peripheral artery disease**	23 (43%)	24 (41%)	0.060
**History of AF**	12 (22%)	13 (22%)	0.543
**Medication**			
AAS	31 (57%)	35 (59%)	0.494
Statins	26 (48%)	30 (51%)	0.461
ACEI/ARB	23 (43%)	20 (34%)	0.399
**ESRD causes**			0.749
Diabetes	21 (39%)	28 (47%)	
Atherosclerosis	6 (10%)	3 (6%)	
GN/FSH	13 (24%)	9 (15%)	
Other	14 (26%)	19 (32%)	

Data are expressed as number (percentage) or mean (standard deviation) accordingly.

AAS = aspirin; ACEI = angiotensin II conversion enzyme inhibitors; AF = atrial fibrillation; ARB = angiotensin II blockers; BMI = body mass index; CAD = coronary artery disease; ESRD = end-stage renal disease; FSH = focal and segmental hyalinosis; GN = glomerulonephritis; HD = low-flux hemodialysis; HDF = on-line hemodiafiltration.

### Dialysis data

Prior to randomization, all patients had previously been treated with conventional hemodialysis 3 times per week, during 4 (IQR 3.5–4.0) hours for at least 2 months. Dialysis vintage, vascular accesses, median Kt/V and UF were comparable (Table 2). Qb was 393 mL/min (IQR 371–436) in the HDF group vs. 360 mL/min (345–384) in the HD group (p<0.001), which might be explained by a higher percentage of catheter in the HD group (37% vs. 24%).

**Table 2:** Dialysis data.

Variables	HD		HDF		p-value
	Baseline	1 year	Baseline	1 year	(baseline)
**Dialysis vintage (months)**	27 (12–53)		21 (7–66)		0.769
**Vascular access**					0.265
*Catheter*	22 (37%)		13 (24%)		
*Native fistula*	34 (58%)		36 (67%)		
*PTFE graft*	3 (5%)		5 (9%)		
**Sessions per week**	3 (3–3)	3 (3–3)	3 (3–3)	3 (3–3)	0.159
**Time per session**	4.0 (3.5–4.0)	4.0 (3.5–4.0)	4.0 (3.5–4.0)	4.0 (3.5–4.0)	0.737
**Qb (ml/min)**	360 (345–384)	379 (350–395)	393 (371–436)	420 (389–455)	<0.001
**UF (L/session)**	3.20 (1.96–3.70)	2.96 (2.50–3.58)	2.86 (1.57–3.68)	2.79 (2.37–3.12)	0.426
**Convective volume (L)**				28.6 (26.2–31.4)	
**Kt/V**_**urea**_	1.48 (1.37–1.62)	1.52 (1.36–1.68)	1.57 (1.38–1.90)	1.91 (1.66–2.22)	0.079
β2M reduction rate* **(%)**				69.2 (65.2–72.8)	

Data are expressed as number (percentage) or median (interquartile range) accordingly.

β2M = β2-microglobulin; HD = low-flux hemodialysis; HDF = on-line hemodiafiltration; PTFE = polytetrafluoroethylene (synthetic fistula); Qb = blood flow; UF = ultrafiltration.

*Values calculated using Bergstrom’s formula

At one year, Kt/V was 1.91 (IQR 1.66–2.22) in HDF vs. 1.52 (1.36–1.68) in HD (p<0.001). In the HDF group, convection volume was 28.6 L (IQR 26.2–31.4), and beta-2-microglobulin reduction rate was 69.2% (IQR 65.2–72.8) at 1-year follow-up.

### Evolution of Hs-TnT, CKMB-mass and other inflammatory markers

**Fig 2:**
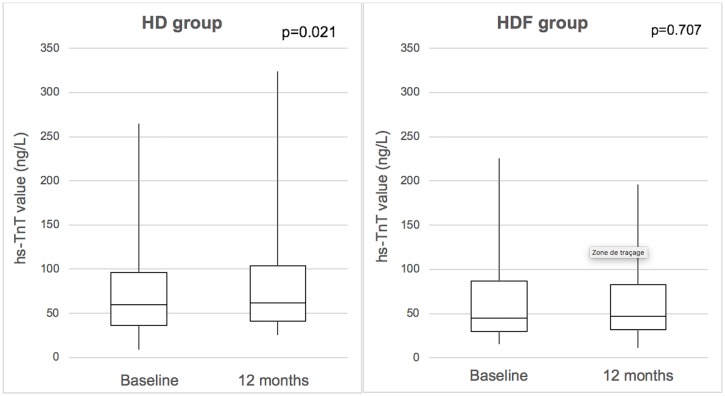
Evolution of Hs-TnT values.

**Fig 3:**
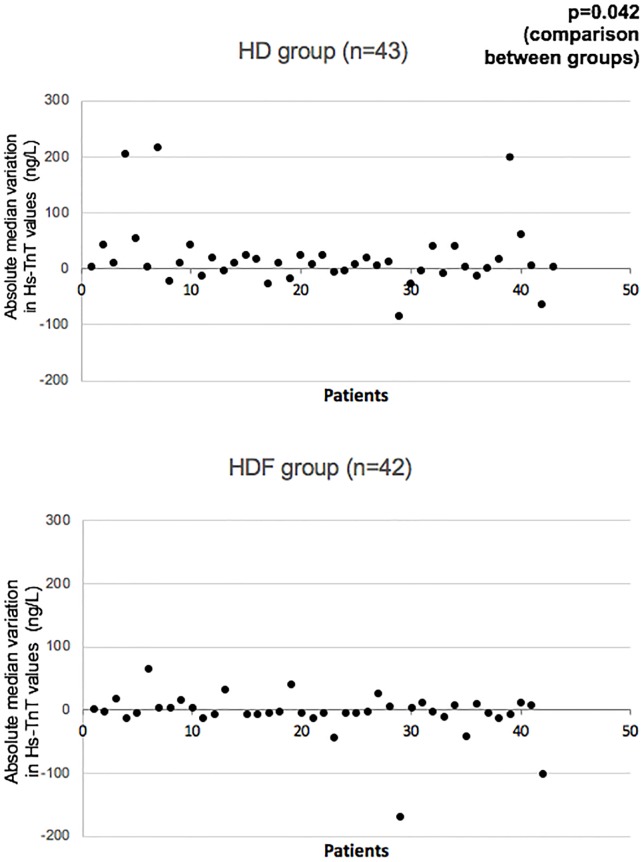
Hs-TnT values variation (delta) from baseline to 1-year follow-up. Hs-TnT values variation is calculated as hs-TnT value at 1-year minus hs-TnT value at baseline. Negative values represent a decrease in hs-TnT over time, whereas positive values indicate an increase. Every point on the graph represents a patient.

At baseline, median hs-TnT value was 49 ng/L (IQR 31–89) in the HDF group vs. 60 ng/L (36–96) in the HD group (p = 0.370). At 1-year follow-up, median hs-TnT remained stable at 49 ng/L (IQR 32–89) in the HDF group (p = 0.707 vs. baseline), but increased to 62 ng/L (40–104) in the HD group (p = 0.021 vs. baseline), which was statistically significant (Table 3, Fig 2). The absolute median variation (delta) in hs-TnT values was calculated as hs-TnT values at 1-year follow-up minus hs-TnT values at baseline. Median variation (delta) in hs-TnT values was -3 ng/L (IQR -7–+8), and +8 ng/L (-5–+25) compared to baseline in the HDF and HD group, respectively (p = 0.042) (Fig 3). Median CKMB-mass value was 2.5 μg/L (IQR 1.7–3.5) at baseline in the HDF group and 2.4 μg/L (1.9–3.4) in the HD group (p = 0.888). At 1-year follow-up, CKMB showed a mild statistically significant decrease in the HD group (from 2.5 to 2.3 μg/L (1.2–3.5); p = 0.043 vs. baseline) (Table 3), while remaining stable in the HDF group.

**Table 3:** Hs-TnT and inflammatory markers.

Variables	HD			HDF		
	Baseline (n = 59)	1 year (n = 43)	p-value	Baseline (n = 54)	1 year (n = 42)	p-value
**β2M pre-session (mg/L)**	34.2 (20.6–47.4)	37.5 (26.6–49.6)	0.030	29.9 (21.5–49.5)	27.2 (21.5–34.4)	0.001
**Albumin (g/L)**	36 (34–38)	35 (33–38)	0.031	36 (34–38)	36 (33–39)	0.074
**Hs-TnT (ng/L)**	60 (36–96)	62 (40–104)	0.021	49 (31–89)	49 (32–89)	0.707
**CKMB-mass (ng/L)**	2.4 (1.9–3.4)	2.3 (1.2–3.5)	0.043	2.5 (1.7–3.5)	2.5 (1.4–3.6)	0.163

Data are expressed as number (percentage) or median (interquartile range) accordingly.

β2M = β2-microglobulin; CKMB-mass = creatine kinase MB-mass; HD = low-flux hemodialysis; HDF = on-line hemodiafiltration; Hs-TnT = High-sensitivity troponin-T; TnI = troponin-I.

One patient in the HDF group had extreme values of hs-TnT (870 ng/L) at 1-year follow-up. However, CKMB-mass at the same time point did not show any elevation. As those results were obtained retrospectively from blood samples kept for further analysis, no investigation based on this extreme hs-TnT value was done at the 1-year time point since it was not known at the time. Upon retrospective review of the medical file, no acute cardiac event had been diagnosed, although dialysis notes reported some shortness of breath which improved with a decrease of the targeted dry weight. Echocardiography done as part of the study (at 1-year follow-up) did show an important decrease in LVEF at the time. This echography report, initially done as part of the study, did result in investigation for this patient in the form of a coronary angiogram, which did not show atherosclerotic coronary lesions important enough to explain the echocardiography findings. Either timing of the event or better sensitivity of troponins might explain why CKMB remained stable while troponins T showed extreme elevation. As this event was not diagnosed as a cardiac event at the time and is a presumption from the laboratory result made retrospectively, we decided to include this subject’s data in the statistical analysis, but it was excluded from the figures to allow better visual assessment of the data.

Albumin values were similar in both groups at baseline and remained stable at 1-year follow-up in HDF, and showed a statistically significant decrease in HD (p = 0.031). More importantly, pre-session beta-2-microglobulin values increased from 34.2 mg/L (IQR 20.6–47.4) at baseline to 37.5 mg/L (IQR 26.6–49.6) in the HD group (p = 0.030), whereas it decreased from 29.9 mg/L (21.5–49.5) to 27.2 mg/L (21.5–34.4) in the HDF group (p = 0.001) (Table 3).

### Echocardiographic data

Echocardiographic data was available for 45 patients in both groups. However, LVMI was irretrievable for 3 patients who had undergone echocardiography in both groups. In the HDF group, median LVEF was 60.0% (55.0–65.0) at baseline and 65.0% (60.0–65.5) at 1-year follow-up (p = 0.040) compared to 60.0% (IQR 55.0–65.0) at baseline and 65.0% (55.0–65.0) at 1-year follow-up (p = 0.312) in the HD group. Median LVMI was 104.3 g/m^2^ (IQR 81.3–125.0) at baseline and 99 g/m^2^ (78.0–124.0) at 1-year follow-up (p = 0.295) in the HDF group, compared to 110.0 g/m^2^ (IQR 87.2–130.5) at baseline and 104.0 g/m^2^ (84.5–141.5) at 1-year follow-up in the HD group (p = 0.421) (Table 4).

**Table 4:** Echocardiographic data.

Variables	HD			HDF		
	Baseline (n = 59)	1 year (n = 45)	p-value	Baseline (n = 54)	1 year (n = 45)	p-value
**LVEF (%)**	60.0 (55.0–65.0)	65.0 (55.0–65.0)	0.312	60.0 (55.0–65.0)	65.0 (60.0–65.5)	0.040
**LVMI (g/m**^**2**^**)**	110.0 (87.2–130.5)	104.0 (84.5–141.5)	0.421	104.3 (81.3–125.0)	99 (78.0–124.0)	0.295

Data are expressed as median (interquartile range)

HD = low-flux hemodialysis; HDF = on-line hemodiafiltration; LVEF = left ventricular ejection fraction; LVMI = left ventricular mass index

### Predictors of Hs-TnT levels at 12 months

Multivariable analyses were done to identify predictors of hs-TnT levels and variation at 12 months in stable patients. The previously mentioned patient was excluded from multivariable analysis since we assumed an acute event happened at the time of the 1-year follow-up. The only statistically significant factors identified as independent predictors of hs-TnT at 12 months were previous coronary artery disease, baseline hs-TnT values and type of dialysis (Table 5). The only statistically significant factors identified as independent predictors of hs-TnT variation were previous coronary artery disease and type of dialysis. Age, gender, diabetes, LVEF at 12 months, Kt/V at 12 months, AAS and statin treatment were not significant predictors of both hs-TnT values and variation at 1-year follow-up.

**Table 5:** Predictors of hs-TnT levels at 12 months (n = 84).

Variables	Univariable		Multivariable		
	ß* (95% CI)	p-value	ß* (95% CI)		p-value
**Age**	0.1 (-0.8–1.0)	0.786	…		
**Gender (reference = male)**	33.0 (4.9–62.1)	0.022	8.1 (-16.7–32.8)		0.516
**CAD**	43.4 (17.1–69.8)	0.002	31.0 (8.3–55.3)		0.009
**Type of dialysis (reference = HD)**	-28.9 (-56.2– -1.7)	0.038	-32.0 (-58.7 –-5.3)		0.014
**Diabetes**	11.5 (-16.4–39.3)	0.416	…		
**AAS**	10.2 (-18.9–39.3)	0.488	…		
**Statin**	5.7 (-22.2–33.7)	0.684	…		
**Baseline hs-TnT level**	0.8 (0.6–1.0)	<0.001	0.7 (0.4–0.9)	<0.001	
**LVEF at 12 months**	-2.2 (-3.7–0.7)	0.004	-0.3 (-1.6–1.0)	0.656	
**Kt/V at 12 months**	-15.8 (-58.6–26.9)	0.464	…		

AAS = aspirin; CI = confidence interval; HD = low-flux hemodialysis; LVEF = left ventricular ejection fraction.

* Beta coefficients (ß) are the standardized estimates from the regression analysis so that the variances of dependent and independent variables are 1.

## Discussion

Although many studies have failed to demonstrate a significant benefit of HDF over HD on all-cause and cardiovascular mortality, there is more and more evidence linking higher convection volumes to benefits on mortality, as was shown in a randomized study by Maduell [6] and post-hoc analysis of other randomized clinical trials [4–5]. More recently, Nubé and al. showed the difference in mortality between HDF and HD to be mainly due to cardiac causes, and reported lower all-cause and cardiovascular mortality risks when higher convection volume were achieved [27].

Many hypothesis have been evoked to explain the better cardiovascular outcomes observed in HDF over HD: less frequent small repeated ischemic injuries through better hemodynamic stability during treatment, ultrafiltration more easily achieved (thus less cardiac workload and hypervolemia), better inflammatory profiles achieved by ultrapure solutions used, cooling effect through high cool volumes being reinjected, etc. Moreover, HDF has been associated with better inflammation profiles, clearance of middle molecules, phosphorus balance and hemodynamic stability [28–30]. Pianta and al. also raised the hypothesis that changes in the uremic milieu could alter the circulating pool of troponin and that changes in physiological factors (such as reduced myocardial stretch) could reduce the release of troponins [15]. Furthermore, in animal studies, some investigators have shown that autoantibodies are produced against circulating troponins I and T, and when present, can contribute to progression to heart failure [31]. In this context, lower levels of hs-TnT (either through better clearance or less release of troponins by myocytes) could be associated with better cardiovascular outcome.

In this study, we showed that, when sufficient convection volumes were obtained, hs-TnT, a strong marker of cardiovascular injury and outcome, remained stable at 1-year follow-up in HDF, but had a tendency to increase in HD. Hs-TnT variation at 12 months was also significantly different between the two modalities: hs-TnT tended to decrease in the HDF group (delta = -3 ng/L (IQR -7-+8)), but to increase in the HD group (delta = +8 ng/L (-5 -+25)). CKMB-mass, however, remained stable in the HDF group, but decreased mildly (median decrease of 0.1 ng/L) in the HD group at 1-year follow-up. Owing to its molecular weight (82 kD), CKMB-mass is not cleared by either HD or HDF, which makes this finding quite surprising. This finding may be attributable to CKMB’s lack of sensitivity to cardiac health as it has now been supplanted by troponins in daily clinical practice. Albumin levels (molecular weight 66 kDa) also showed a peculiar course over the follow-up period which might reflect greater inflammation in the HD group.

Studies have also shown that decreased LVEF and increased LVMI are mortality and cardiovascular disease risk factors in ESRD patients [32]. LVMI regression and stabilisation or increase in LVEF could improve prognosis for those patients. In our HDF group, despite the best efforts to maintain high convection volumes throughout the study duration, LVMI did not regress. However, there is the impression that HDF may be linked to an increased LVEF at 1-year follow-up as our study showed statistically significant changes in LVEF in the HDF group at 1-year follow-up, whereas changes in the HD group were not statistically significant. The CONTRAST study also failed to show significant changes on LVMI and LVEF with HDF compared to HD over time. Nevertheless, they observed a trend toward increased LVMI in HD compared to stability in HDF, and decreased LVEF in HD compared to stability in HDF [7]. Rodriguez Castellanos and al. also found a significant increase in LVEF and a trend to smaller increase in LVMI only in their HDF group over time [21].

In our study, multivariable analysis showed coronary artery disease to be a significant predictor of hs-TnT values and variation at 1-year follow-up. Moreover, modality of treatment (HDF vs. HD) was also a significant predictor of hs-TnT values at 1-year follow-up. Although previous studies in the chronic renal insufficiency population have shown older age, LV mass and diabetes to be associated with higher hs-TnT values [13], our study failed to show these factors as predictors for hs-TnT values and variation at 1-year follow-up. Interestingly, other studies have also linked elevated hs-TnT values to a history of coronary artery disease, along with peripheral vascular disease [15] and LVEF <50% in clinically stable hemodialysis patients [16]. The small number of patients in our study might have an impact on which factors were identified as more significant predictors of hs-TnT values and variation. It should also be noted that echocardiography were done less than 24 hours after a dialysis session, whereas blood sampling was done pre-dialysis. Echocardiography were done after dialysis as it is well known that results are intricately correlated with volume status. Hs-TnT values are also influenced by volume overload but our main focus here was the long term changes and not variation pre and post dialysis. In our study, we did not make any conclusions on correlation between troponin values and echocardiography data. However, we did include LVEF at 12 months in our multivariable analysis looking at predictors for hs-TnT values and variation at 1-year follow-up, which failed to show statistically significant results for LVEF.

Unfortunately, dry weight changes, ultrafiltration values per treatment session and hemodynamic status (blood pressure values before, during and after treatment) were not recorded for the purpose of this study, although it was noted as per usual clinical practice in the dialysis unit. However, changes in the electronic software used on the dialysis unit made retrieving such values quite difficult and would yield a significant number of missing values, making the statistical analysis unreliable. Furthermore, no other markers of fluid status were recorded as part of this study (ie. cardiothoracic index from chest X ray or bioimpedance measurements), making it more difficult to interpret the results according to volume status. Therefore, those parameters could not be evaluated as predictors of hs-TnT values and variation at 1-year follow-up.

Whether hs-TnT variation with HDF, compared to HD, correlates with better clinical outcomes, such as mortality and cardiovascular events, is not known. Unfortunately, due to the small number of patients included in this sub-study, the number of deaths, cardiovascular events or hospitalization were too small to make any conclusion on the occurrence of such events related to dialysis modality or troponin values. Also, whether HDF might benefit more to some patients than others according to their cardiovascular risk is still unknown.

Baseline hs-TnT values reported in our study are similar to previous studies showing elevated baseline values in the stable chronic dialysis population, ranging from 34 (Fahim [33]) to 63 ng/L (Wolley [16]) and associating higher levels to mortality. Moreover, data are still conflicting on the evolution of troponins during hemodialysis. Assa and al. reported rises in troponins I during hemodialysis, suggesting that hemodialysis has an acute deleterious effect on the heart [34]. However, Cardinaels and al. demonstrated a significant reduction in hs-TnT, hs-TnI and NTproBNP following HDF, especially following 8-hours sessions [35]. Wolley and al. also reported that hs-TnT decreased by an average of 16% over a hemodialysis session. Owing to its 39,7 kDa molecular weight, troponin T leakage and adsorption of troponin degradation products by high-flux membranes could be potential mechanisms [16]. Furthermore, convective clearance by HDF might also explain those reductions over time. Our study did not intend to evaluate the hs-TnT variation over a single session, but to determine their evolution over time. Blood samples were always drawn before a dialysis session. Therefore, such variation over a session should not interfere with the results of our study. However, hs-TnT would need to be measured on dialysate samples to evaluate if these reductions are due to clearance.

Our study has important strengths. Patients were initially randomized as part of the CONTRAST study. Although some patients had to be excluded from the present study, patients’ characteristics and dialysis data remained comparable between both groups. Also, both the biochemist who tested the blood samples and the cardiologists who evaluated the patients were blinded to the treatment assignment. Moreover, high convection volumes were achieved throughout the study. Consequently, comparable convection volumes must be used for our results to be applicable.

Our study also has many limitations. We only evaluated a small number of patients, as some patients had to be excluded because of missing data (6 in the HD group vs. 11 in the HDF group). Loss to follow-up also decreased the number of patients who could be evaluated at 1-year follow-up. Thus, attrition bias could influence the result of this study. Evolution of cardiac function over time and cardiovascular events are complex and multifactorial issues. Unfortunately, many aspects of anemia management, iron status, nutritional status, intradialytic tolerance (arrhythmia, intradialytic hypotension), fluid management and volume status (ultrafiltration, dry weight changes, other biomarkers) were not explored in this study which is also a limitation of our findings as we could not correlate troponin values and echocardiography measurements to such factors.

To our knowledge, this study is the first to evaluate the evolution of hs-TnT at 1-year follow-up in HDF with high convection volumes compared to HD, showing stability in hs-TnT values with HDF whereas associating low-flux HD with a statistically significant increase in hs-TnT levels. Our study is a modest effort in identifying biological markers who could be associated with the clinical benefit observed in previous trials, in a subcohort of patients for whom high convection volume HDF was achieved throughout follow-up. Future studies are needed to establish whether stability of hs-TnT values is linked to better outcome in HDF patients. Studies on this matter should investigate all pertinent aspects involved in cardiovascular outcomes, including volume status and fluid management, intradialytic tolerance, anemia management and iron status, as well as nutritional aspect and comparison of other cardiac biomarkers or imaging studies. Furthermore, measurements of cardiac biomarkers in the dialysate over multiple sessions would also be informative to distinguish between the clearance of such biomarkers and an actual benefit of treatment modality on myocardial injury.

## Supporting information

S1 File Anonymized data set. (XLSX)

S1 Checklist CONSORT guidelines checklist. (DOC)

S1 Protocol Cost-effectiveness study protocol—English version. (DOCX)

S2 Protocol Cost-effectiveness study protocol—French version. (DOCX)
