# Supplementary material for: Evolution of high-sensitivity troponin-T and echocardiography parameters in patients undergoing high efficiency on-line hemodiafiltration versus conventional low-flux hemodialysis
Source: PLoS One. 2019 Oct 22;14(10):e0223957. doi: 10.1371/journal.pone.0223957 (PMC6804981; doi:10.1371/journal.pone.0223957)
Supplement: S1 Protocol — (DOCX) [file pone.0223957.s003.docx]

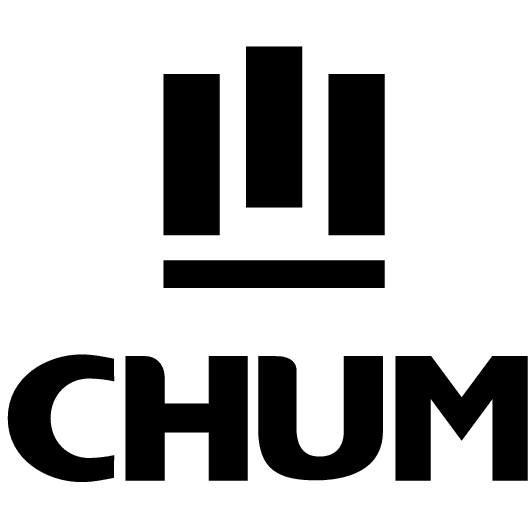


STUDY PROTOCOL HDF

**(post CONTRAST study)**

**ORIGINAL TITLE :** « Étude prospective randomisée comparant l’hémodiafiltration en ligne et l’hémodialyse conventionnelle d’un point de vue économique et pharmaco-économique »

**TITLE (translated) : “Randomised prospective study comparing on-line hemodiafiltration and conventional hemodialysis from an economic and pharmaco-economic point of view”**

**INVESTIGATOR :** Dr Renée Lévesque, nephrologist

**Introduction :**

Interest is growing about on-line hemodiafiltration (HDF) as a standard treatment method for end-stage kidney disease. HDF is still not widespread in North America, mainly for economic reasons, but has been used for over 30 years in Europe.

It is currently the most beneficial method of dialysis treatment. Compared to other dialysis modalities, HDF allows the most complete and efficient purification, it is better tolerated hemodynamically, the use of ultrapure solutions decreases the circulating levels of biomarkers of inflammation and the pressure profile inside the dialyzer prevents transport of contaminants back to the patient's blood (1-3).

Compared to diffusive dialysis strategies that essentially purify small molecules such as urea and creatinine, HDF combines both diffusion and convection, thus allowing purification of both small and medium molecules (MM) (up to 30-40 kDa). Clinical studies have shown that beta-2-microglobulin (beta2m), a 11.8kDa MM, unable to cross a low-flux membrane (conventional hemodialysis (HD)), is effectively removed in HDF and that levels pre-dialysis are lowered in the long course (4-5). Similarly, MM purification such as "advanced glycation end-products" (AGE), leptin, and complement factor D, is increased by convective transport (4-6). It has also been shown that on-line HDF further lowers phosphorus levels than standard HD (4-5). Finally, several recent studies show an improvement in the micro-inflammatory state and endothelial function (7-10), an improvement in nutritional status, as well as a decrease in the need for erythropoietin required to maintain a stable hematocrit (10-11) with HDF.

It is assumed that by allowing the purification of medium molecules, HDF could reduce cardiovascular risk compared to standard HD (12). Recently, observational studies have reported better HDF survival rates and a 35-59% reduction in mortality when using high-efficiency HDF (13-15). Some multicenter randomized controlled trials are underway, including the CONTRAST study, which compares HDF with conventional HD in terms of morbidity and all-cause / cardiovascular mortality (16). The CONTRAST study ended on 01-01-2011 and the results should be available in the coming months.

While awaiting these results and whatever the conclusions of this study, we can always argue that HDF is a treatment that costs a little more than conventional HD. Several experts have evaluated the cost difference compared to conventional HD, expressing it in "additional costs compared to conventional HD" (17). They came up with results that are still pretty similar and around $ 2000/ patient-year or 15-25 Euros/week. However, this comparison only takes into account the direct costs of the technique (equipment, consumables) whereas a true comparative study should also take into consideration both direct and indirect costs of dialysis: estimated costs of difference in quality of life, in survival, in days of hospitalization, in the occurrence of cardiovascular events, in accessibility/productivity at work, in the cost of medication, etc. No study presenting a rigorous methodology and taking into account all the costs generated by the different dialysis techniques has been identified (17).

The additional cost attributed to HDF may in fact represent savings compared to conventional HD or other dialysis modalities, and might be a false limitation to the expansion of the technique.

**PROTOCOL AND OBJECTIVES :**

The place of conduct of the clinical trial will be the Hôpital St-Luc (HSL) site of the CHUM. This is a prospective randomized study that will compare the 2 treatment modalities, HD and HDF, via economic and pharmacoeconomic parameters.

**The primary objectives of the study are:**

- comparing the cost of the drug bill between the 2 groups (HD and HDF);

- discriminate a reduction in the costs of erythropoietin in HDF for the same control of anemia compared to the HD group;

- show a decrease in chelator costs in HDF for the same control of the phosphocalcic balance.

**The secondary objectives are:**

- to highlight a decrease in erythropoietin requirements and an easy control of anemia in the HDF group;

- to show a decrease in the need for chelators in the HDF group for the same control of the phosphocalcic balance;

- to highlight a decrease in hospital stays and related costs in the HDF group

- to verify if there is a stabilization of the left ventricular mass (or regression of left ventricular hypertrophy) in the HDF group compared to the HD group.

Patients will be followed for 3 years. Treatments will be comparable in terms of duration and frequency of sessions. A set of demographic and clinical data will be collected from patients' medical records and throughout the study.

All events will be noted (hospitalizations, reasons, duration) and the list of drugs will be compiled every 3 months. This information will eventually be analyzed for the purpose of economic comparison of the two treatment modalities.

The biochemical data that is part of the usual work-up of the dialysis patients will be collected every month, as well as the information concerning the annual cardiac echocardiography of the patients will be preserved and analyzed.

**Recruitment :**

All patients already randomized in the CONTRAST study since 2007 (1:1) will remain in their respective group if they agree to continue the observation. Initially, a total of 80 patients had been randomized. Many died, moved or were grafted. There are currently about 50 patients among the 80 initially recruited. A consent form will be presented to them.

The other subjects in this study will be recruited from the CHUM HSL dialysis patient population provided that they meet the established criteria that are the same as those prevailing for the CONTRAST study, and provided they also consent to participate in the study.

**Inclusion criteria:**

• Patients at least 18 years old.

• Patients able to understand the protocol and give informed consent.

• Stable patients treated with hemodialysis for ≥ 8-12 hours/week (2-3 sessions) for at least 2 months, with a Kt/V≥ 1.2.

**Exclusion criteria:**

• Severe non-compliance (repeated absences, dialysis sessions frequently shortened without valid reason, severe non-compliance with fluid restriction, non-compliance with prescribed medication regimens).

• Life expectancy ≤ 3 months of non-renal cause.

• High-flux HD treatment in the previous 6 months.

**Number of patients to recruit**

We have 7 HDF stations for up to 42 patients to be treated with this technique. The study therefore aims to fill the positions and a total of 80-84 active patients to be followed in the study. The places that will be left vacant during the study (death, transplant, relocation) will be filled by other randomized patients.

The randomization of new patients will be done with a 1:1 ratio (on-line hemodiafiltration vs conventional hemodialysis). The randomization process will be totally independent of the patient recruitment process. A person not related to the study will be responsible for generating the randomization sequence (blocks of variable size). After obtaining consent, the recruiter will contact the Randomization Sequence Manager who will indicate to which group the next subject is assigned.

**Stabilization period / washout**

New patients added to the group should be stabilized on hemodialysis for a period of at least two months before they can be randomized.

**Routine care**

Treatments will be comparable in terms of duration and frequency of sessions. Patients will be subjected to the same blood tests as those previously used. The metabolic control of patients will be maintained according to the current recommendations. Antihypertensive medication, hypolipidemic medication, medication used to treat anemia and renal osteodystrophy will also be prescribed according to these recommendations, and in the absence of recommendation, according to usual care.

At the beginning of the study and once a year thereafter, a larger amount of blood will be collected to measure markers of endothelial dysfunction, chronic inflammation, atherosclerosis, oxidative stress and apoptosis. Once a year, patients will complete a quality of life questionnaire and undergo an assessment of their nutritional status. Like all patients on dialysis, subjects will be submitted once a year to a cardiac ultrasound. This imaging test will be performed within 24 hours of dialysis treatment in the middle of the week, when the patient is less than 1 kg from his dry weight.

A sufficient dialysis dose should be reached (spKt/V of at least 1.2 per treatment in HD and HDF, and a purification of b2M of at least 60% in HDF).

Patients randomized to online HDF will be treated in post-dilution mode as standard (unless contraindicated). The minimum reinjection dose targeted will be 100ml/min or 6L/hour (double in case of temporary passage in pre-dilution). The other parameters/specifications (membranes, dialysis parameters, online technique) will be the same as with the CONTRAST study and are considered standard in HDF.

# Statistics

We performed a power calculation to determine whether the expected sample was large enough to detect a difference of $CAN2,000 in drug costs between the 2 groups (in favor of HDF). This difference was chosen because it represents the 'extra cost' related to HDF in terms of technique, as discussed above. To do this, we analyzed the distribution of drug costs at the beginning of the CONTRAST study in subjects already recruited. The standard deviation of the variable was $CAN780.00. Using 40 subjects per group and an alpha error of 0.05, we obtained 100% power for detecting a difference of $CAN2000.00 or more between the 2 groups, confirming the feasibility of the project.

As our primary endpoint is the difference in drug costs between the 2 groups at 3-year follow-up, we will examine the distribution of this variable. If it is normally distributed, we will perform a two-tailed Student test for independent samples. Otherwise, we will perform a logarithmic transformation of the variable. If this does not restore to normality, we will perform a Mann-Whitney test.

We will analyze the other objectives in the same way, since the measurements made are all continuous variables. For the comparison between indexed ventricular masses, we will calculate the difference between the pre-randomization ventricular masses and 3 years after the start of the study for each subject. This difference will be compared between the 2 groups using a bilateral Student's independent samples Test.

**Conclusion**

So far, conventional HD remains the standard treatment for chronic hemodialysis patients. Online HDF is the most effective mode of extra-renal cleansing, in addition to reducing the chronic inflammatory state responsible for long-term complications (undernutrition, accelerated atherosclerosis, significant cardiovascular morbidity/mortality). Some randomized controlled trials are underway to see whether the undeniable benefits of HDF also translate into a reduction in patient mortality, which could dramatically change the overall prognosis for patients and ensure that HDF becomes the reference dialysis modality.

The additional cost of the technique compared to conventional hemodialysis is the last stumbling block to be overcome and could be a very relative disadvantage largely offset by a reduction in societal costs.

**References :**

1. Locatelli F et al. Hemodiafiltration – A new Era. Contrib Nephrol. Basel, Karger, 2011, vol 168, pp 5-18.
2. Canaud B et al. Hemodiafiltration, state of the art. Nephrol Dial Transplant 1998; 13 Suppl 5 :3-11.
3. Ledebo I, Blankestijn PB. Haemodiafiltration – optimal efficiency and safety. Nephrol Dial Transplant plus 2009; 1-9.
4. BlankesRabindraneth K et al. Compaison of hemodialysis, hemofiltration and acetate-free biofiltration for ESRD. Am J Kidney Dis 2005; 45 :437-447.
5. Van Laecke et al. Online hemodiafiltration. Artificial Organs. 2006; 30 :579-585.
6. Lin et al. Reduction of advanced glycation end product levels by on-line hemodiafiltration in long-term hemodialysis patients. Am J Kidney Dis. 2003 Sep; 42(3) :524-531.
7. Carracedo J et al. Online hemodiafiltration reduces tre proinflammatory CD14+CD16+ monocyte-derived dentritic cells : a prospective crossover trial. J Am Soc Nephrol 2006; 17 :2315-2321.
8. Aires I et al. Online hemodiafiltration with high volume substitution fluid : long term efficacy and security. Nephrol Dial Transplant 2006; 21 : 756-762.
9. Malyszko J et al. Markers of endothelial damage in patients on hemodialysis and hemodiafiltration. J. Nephrol 2006; 19 :1504.
10. Vaslaki L et al. Online hemodiafiltration vs hemodialysis : stable hematocrit with less erythropoietin and improvement of other relevant blood parameters. Blood Purif 2006; 24 : 163-173.
11. Fishbach et al. On-line haemodiafiltration : 4 year experience in children. Clin Nephrol 2008; 69 :279-294.
12. Penne EL et al. Resolving controversies regarding hemodiafiltration versus hemodialysis : the Dutch Convective Transport Study. Semin Dial 2005; 18 : 47-51.
13. Canaud B et al. Mortality risk for patients receiving hemodiafiltration versus hemodialysis : European results from the DOPPS. Kidney Int 2006; 69 : 2087-2093.
14. Jirka T et al. The impact of online hemodiafiltration on patients survival : results from a large network database. Nephrol Dial Transplant 2005; 70 : 1524-1525.
15. Bosch JP et al. Clinical use of high-efficiency hemodialysis treatments : long term assessment. Hemodial Int 2006; 10 : 73-81.
16. Penne EL et al. Effect of increased convective clearance by on-line hemodiafiltration on all cause and cardiovascular mortality in chronic hemodialysis patients – the Dutch CONvective TRAnsport STudy (CONTRAST) : rationale and design of a randomised controlled trial. Curr Control Trials Cardiovas Med. 2005 May 20; 6(1) : 8-18.
17. Évaluation de l’hémofiltration et l’hémodiafiltration avec production en ligne du liquide de substition. Rapport de l’Anaes. 2001 fév : 1-86.
