# Supplementary material for: Evolution of high-sensitivity troponin-T and echocardiography parameters in patients undergoing high efficiency on-line hemodiafiltration versus conventional low-flux hemodialysis
Source: PLoS One. 2019 Oct 22;14(10):e0223957. doi: 10.1371/journal.pone.0223957 (PMC6804981; doi:10.1371/journal.pone.0223957)
Supplement: S2 Protocol — (DOCX) [file pone.0223957.s004.docx]

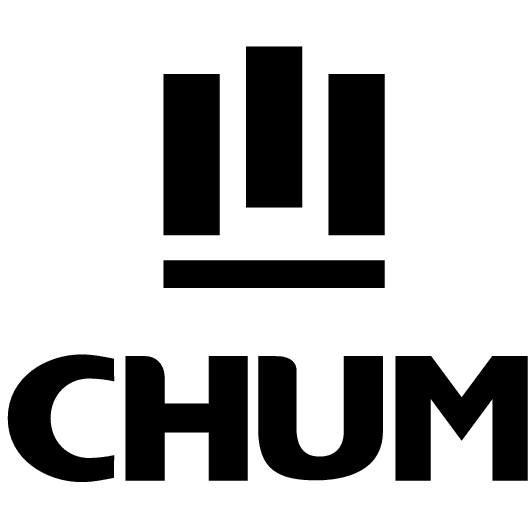


PROTOCOLE DE RECHERCHE HDF

**(post CONTRAST study)**

**TITRE :** « Étude prospective randomisée comparant l’hémodiafiltration en ligne et l’hémodialyse conventionnelle d’un point de vue économique et pharmaco-économique »

**CHERCHEUR :** Dr Renée Lévesque, néphrologue

**Introduction:**

L’intérêt croît pour l’hémodiafiltration en ligne (HDF) comme méthode de traitement standard de l’insuffisance rénale chronique terminale. L’HDF est encore peu répandue en Amérique du Nord, essentiellement pour des motifs économiques, mais elle est utilisée depuis plus de 30 ans en Europe.

Elle constitue actuellement le mode de traitement de dialyse le plus avantageux qui soit. Par rapport aux autres modalités de dialyse, l’HDF permet l’épuration la plus complète et la plus efficace, elle est mieux tolérée hémodynamiquement, l’utilisation de solutions ultrapures diminue les taux circulants des biomarqueurs d'inflammation et le profil de pression à l’intérieur du filtre empêche le transport à rebours des contaminants vers le sang du malade (1-3).

Par rapport aux stratégies de dialyse diffusive qui épurent essentiellement les petites molécules comme l’urée et la créatinine, l’HDF rallie à la fois la diffusion et la convection, permettant ainsi une épuration à la fois des petites et moyennes molécules(MM) (jusqu’à 30-40 kDa). Des études cliniques ont démontré que la beta-2-microglobuline (beta2m), une MM de 11,8kDa, incapable de traverser une membrane à bas flux (hémodialyse (HD) conventionnelle), est éliminée de façon efficace en HDF et que les niveaux pré-dialyse sont abaissés au long cours (4-5). De la même manière, l’épuration de MM comme les « advanced glycation end-products » (AGE), la leptine, et le facteur D du complément, est augmentée par le transport convectif (4-6). Il a aussi été démontré que l’HDF on line abaisse davantage les niveaux de phosphore que l’HD standard (4-5). Finalement, plusieurs études récentes démontrent une amélioration de l’état micro-inflammatoire et de la fonction endothéliale (7-10), une amélioration du statut nutritionnel, ainsi qu’une diminution des besoins d’érythropoïétine requis pour maintenir un hématocrite stable (10-11).

On présume qu’en permettant l’épuration de moyennes molécules, l’HDF pourrait réduire le risque cardiovasculaire par rapport à l’HD standard (12). Récemment, des études observationnelles ont rapporté de meilleurs taux de survie en HDF et une réduction de la mortalité de l’ordre de 35-59% lorsqu’on fait de l’HDF à haute efficacité (13-15). Quelques études multicentriques randomisées contrôlées sont en cours, dont l’étude CONTRAST qui compare l’HDF à l’HD conventionnelle en terme de morbidité et de mortalité toute cause / cardiovasculaire (16). L’étude CONTRAST a pris fin le 01-01-2011 et les résultats devraient être disponibles dans les prochains mois.

En attendant ces résultats et quelles que soient les conclusions de cette étude, on pourra toujours argumenter que l’HDF est un traitement qui coûte un peu plus cher que l’HD conventionnelle. Plusieurs experts ont évalué la différence de coûts par rapport à l’HD conventionnelle, l’exprimant en « surcoûts par rapport à l’HD conventionnelle »(17). Ils sont arrivés à des résultats toujours à peu près similaires et avoisinant les $2000/patient-année ou 15-25 euros/semaine. Toutefois, cette comparaison ne tient compte que des coûts directs de la technique (équipement, consommables) alors qu’une vraie étude comparative devrait aussi prendre en considération à la fois les coûts directs et les coûts indirects de la dialyse : coûts estimés d’une différence dans la qualité de vie, dans la survie, dans les jours d’hospitalisation, dans la survenue d’évènements cardio-vasculaires, dans l’accessibilité / productivité au travail, dans la facture de médicaments, etc. Aucune étude présentant une méthodologie rigoureuse et tenant compte de l’ensemble des coûts engendrés par les différentes techniques de dialyse n’a été identifiée(17).

Et le coût supplémentaire attribué à l’HDF est peut-être en fait une économie par rapport à l’HD conventionnelle ou aux autres modalités de dialyse, et une fausse limitation à l’expansion de la technique.

**PROTOCOLE ET OBJECTIFS :**

Le lieu de la conduite de l'essai clinique sera le site HSL du CHUM. Il s’agit d’une étude prospective randomisée qui va comparer les 2 modalités de traitement, HD et HDF, via des paramètres économiques et pharmaco-économiques.

Les objectifs primaires de l’étude sont les suivants :

-comparer le coût de la facture de médicaments entre les 2 groupes (HD et HDF);

-mettre en évidence une diminution des coûts de l’érythropoïétine en HDF pour un même contrôle de l’anémie par rapport au groupe HD;

-mettre en évidence une diminution des coûts de chélateurs en HDF pour un même contrôle du bilan phospho-calcique.

Et les objectifs secondaires sont :

-Mettre en évidence une diminution des besoins en érythropoïétine et un contrôle de l’anémie facilité dans le groupe HDF ;

-mettre en évidence une diminution des besoins en chélateurs dans le groupe HDF pour un même contrôle du bilan phospho-calcique;

-mettre en évidence une diminution des séjours hospitaliers et des coûts reliés dans le groupe HDF

-vérifier s’il y a une stabilisation de la masse ventriculaire gauche (ou une régression de l’hypertrophie ventriculaire gauche) dans le groupe HDF par rapport au groupe HD.

Les patients seront suivis pendant 3 ans. Les traitements seront comparables quant à la durée et à la fréquence des séances. Un ensemble de données démographiques et cliniques sera colligé à même les dossiers médicaux des patients et tout au long de l’étude.

Tous les événements seront notés (hospitalisations, motifs, durée) et la liste de médicaments sera compilée à chaque 3 mois. Ces informations seront éventuellement analysées à des fins de comparaison économique des 2 modalités de traitement.

Les données biochimiques faisant partie du bilan usuel des patients dialysés seront colligées à chaque mois de même que seront conservées et analysées les informations concernant l’échographie cardiaque annuelle des patients.

**Mode de recrutement :**

Tous les patients déjà randomisés dans l’étude CONTRAST depuis 2007 (1 :1) resteront dans leur groupe respectif s’ils acceptent de poursuivre l’observation. Initialement, un total de 80 patients avait été randomisé. Plusieurs sont décédés, ont déménagé ou ont été greffés. Il reste actuellement une cinquantaine de patients parmi les 80 initialement recrutés. Un formulaire de consentement leur sera présenté.

Les autres sujets de cette étude seront recrutés à partir de la population des patients dialysés de l’HSL du CHUM à condition de satisfaire aux critères établis qui sont les mêmes que ceux qui prévalaient pour l’étude CONTRAST, et à condition également qu’ils consentent à participer à l’étude.

**Critères d’inclusion :**

- Patients âgés d’au moins 18 ans.
- Patients aptes à comprendre le protocole et à donner un consentement éclairé.
- Patients stables traités par hémodialyse à raison de ≥ 8-12 heures / semaine (2-3 séances) depuis au moins 2 mois, avec un Kt/V ≥ 1.2.

**Critères d’exclusion :**

- Non observance sévère (par absences répétées, séances de dialyse fréquemment écourtées sans motif valable, non respect sévère de la restriction liquidienne, non observance des traitements médicamenteux prescrits).
- Espérance de vie ≤ 3 mois de cause non rénale.
- Traitement par HD high-flux dans les 6 mois précédents.

# Nombre de patients à recruter

Nous disposons de 7 postes d’HDF pour un maximum de 42 patients à traiter avec cette technique. L’étude vise donc à ce que les postes soient comblés et qu’un total de 80-84 patients actifs soient suivis dans l’étude. Les places qui seront laissées vacantes en cours d’étude (décès, greffe, déménagement) seront comblées par d’autres patients randomisés.

La randomisation des nouveaux patients se fera avec un ratio 1 :1, hémodiafiltration en ligne vs hémodialyse conventionnelle. Le processus de randomisation sera totalement indépendant du processus de recrutement des patients. Une personne non liée à l’étude, sera chargée de générer la séquence de randomisation (blocs de taille variable). Après l'obtention du consentement, la personne chargée du recrutement contactera le responsable de la séquence de randomisation qui lui indiquera le groupe auquel le prochain sujet est assigné.

**Période de stabilisation / washout.**

Les nouveaux patients qui se rajouteront au groupe devront être stabilisés en hémodialyse pendant une période d’au moins deux mois avant de pouvoir être randomisés.

**Soins de routine**

Les traitements seront comparables en ce qui a trait à la durée et à la fréquence des séances. Les patients seront soumis aux mêmes bilans sanguins que ceux qui prévalaient jusque là de routine. Le contrôle métabolique des patients sera maintenu selon les recommandations en vigueur. La médication anti-hypertensive, hypolipémiante, la médication utilisée pour traiter l’anémie et l’ostéodystrophie rénale seront aussi prescrites selon ces recommandations, et en l’absence de recommandation, selon la routine usuelle.

Au début de l’étude et une fois par année ensuite, une quantité plus grande de sang sera prélevée pour doser des marqueurs de dysfonction endothéliale, d’inflammation chronique, d’athérosclérose, de stress oxydant et d’apoptose. Une fois par année, ils rempliront un questionnaire de qualité de vie et subiront une évaluation de leur statut nutritionnel. Comme tous les patients dialysés, les sujets à l’étude seront soumis une fois l’an à subir une échographie cardiaque. Cet examen d’imagerie sera effectué dans les 24 heures suivant un traitement de dialyse, en milieu de semaine, alors que le patient sera à moins de 1 kg de son poids sec.

Une dose de dialyse suffisante devra être atteinte (spKt/V d’au moins 1.2 par traitement en HD et HDF, et une épuration de la b2M d’au moins 60% en HDF).

Les patients randomisés en HDF en ligne seront traités en post-dilution de façon standard (à moins de contre-indication). La dose de réinjection minimale visée sera de 100ml/min ou 6L/heure (le double en cas de passage temporaire en pré-dilution). Les autres paramètres / spécifications (membranes, paramètres de dialyse, technique en ligne) seront les mêmes qu’avec l’étude CONTRAST et sont considérés comme standard en HDF.

# Statistiques

Nous avons effectué un calcul de puissance afin de déterminer si l'échantillon prévu était assez important pour détecter une différence de 2000$ canadiens en terme de coûts des médicaments entre les 2 groupes (en faveur de l'HDF). Cette différence a été choisie puisqu'elle représente le 'surcoût' lié à l'HDF en terme de technique, tel que discuté plus haut. Pour ce faire, nous avons analysé la distribution de coûts des médicaments au début de l'étude Contrast chez les sujets déjà recrutés. L'écart-type de la variable était de 780.00$ canadiens. En utilisant 40 sujets par groupes et une erreur alpha de 0.05, nous avons obtenu une puissance de 100% pour la détection d'une différence de 2000.00$ canadiens ou plus entre les 2 groupes, confirmant la faisabilité du projet.

Notre critère de jugement primaire étant la différence en terme de coûts liés aux médicaments entre les 2 groupes à 3 ans, nous examinerons la distribution de cette variable. Si elle est normalement distribuée, nous effectuerons un test de Student bilatéral pour des échantillons indépendants. Dans le cas contraire, nous effectuerons une transformation logarithmique de la variable. Si cela ne restore pas la normalité, nous effectuerons un test de Mann-Whitney.

Nous analyserons les autres objectifs de la même façon, puisque les mesures effectuées sont toutes des variables continues. Pour ce qui est de la comparaison entre les masses ventriculaires indexées, nous calculerons la différence entre les masses ventriculaires pré-randomisation et 3 ans après le début de l'étude pour chaque sujet. Cette différence sera comparée entre les 2 groupes à l'aide d'un test de Student bilatéral pour échantillons indépendants.

**Conclusion**

Jusqu’à présent, l’HD conventionnelle demeure le traitement standard des hémodialysés chroniques. L’HDF en ligne constitue le mode d’épuration extra-rénal le plus efficace qui soit, en plus de réduire l’état inflammatoire chronique responsable des complications au long cours (dénutrition, athérosclérose accélérée, morbidité/mortalité cardio-vasculaire importante). Quelques études randomisées contrôlées sont en cours pour voir si les avantages indéniables de l’HDF se traduisent aussi par une réduction de la mortalité des patients, ce qui pourrait modifier considérablement le pronostic global des patients et faire en sorte que l’HDF devienne le mode de dialyse de référence.

Le surcoût de la technique par rapport à l’hémodialyse conventionnelle constitue la dernière pierre d’achoppement à abattre et pourrait n’être qu’un désavantage bien relatif largement compensé par une réduction des coûts de société.

**Références :**

1. Locatelli F et al. Hemodiafiltration – A new Era. Contrib Nephrol. Basel, Karger, 2011, vol 168, pp 5-18.
2. Canaud B et al. Hemodiafiltration, state of the art. Nephrol Dial Transplant 1998; 13 Suppl 5 :3-11.
3. Ledebo I, Blankestijn PB. Haemodiafiltration – optimal efficiency and safety. Nephrol Dial Transplant plus 2009; 1-9.
4. BlankesRabindraneth K et al. Compaison of hemodialysis, hemofiltration and acetate-free biofiltration for ESRD. Am J Kidney Dis 2005; 45 :437-447.
5. Van Laecke et al. Online hemodiafiltration. Artificial Organs. 2006; 30 :579-585.
6. Lin et al. Reduction of advanced glycation end product levels by on-line hemodiafiltration in long-term hemodialysis patients. Am J Kidney Dis. 2003 Sep; 42(3) :524-531.
7. Carracedo J et al. Online hemodiafiltration reduces tre proinflammatory CD14+CD16+ monocyte-derived dentritic cells : a prospective crossover trial. J Am Soc Nephrol 2006; 17 :2315-2321.
8. Aires I et al. Online hemodiafiltration with high volume substitution fluid : long term efficacy and security. Nephrol Dial Transplant 2006; 21 : 756-762.
9. Malyszko J et al. Markers of endothelial damage in patients on hemodialysis and hemodiafiltration. J. Nephrol 2006; 19 :1504.
10. Vaslaki L et al. Online hemodiafiltration vs hemodialysis : stable hematocrit with less erythropoietin and improvement of other relevant blood parameters. Blood Purif 2006; 24 : 163-173.
11. Fishbach et al. On-line haemodiafiltration : 4 year experience in children. Clin Nephrol 2008; 69 :279-294.
12. Penne EL et al. Resolving controversies regarding hemodiafiltration versus hemodialysis : the Dutch Convective Transport Study. Semin Dial 2005; 18 : 47-51.
13. Canaud B et al. Mortality risk for patients receiving hemodiafiltration versus hemodialysis : European results from the DOPPS. Kidney Int 2006; 69 : 2087-2093.
14. Jirka T et al. The impact of online hemodiafiltration on patients survival : results from a large network database. Nephrol Dial Transplant 2005; 70 : 1524-1525.
15. Bosch JP et al. Clinical use of high-efficiency hemodialysis treatments : long term assessment. Hemodial Int 2006; 10 : 73-81.
16. Penne EL et al. Effect of increased convective clearance by on-line hemodiafiltration on all cause and cardiovascular mortality in chronic hemodialysis patients – the Dutch CONvective TRAnsport STudy (CONTRAST) : rationale and design of a randomised controlled trial. Curr Control Trials Cardiovas Med. 2005 May 20; 6(1) : 8-18.
17. Évaluation de l’hémofiltration et l’hémodiafiltration avec production en ligne du liquide de substition. Rapport de l’Anaes. 2001 fév : 1-86.
